# *In vitro* induction of NETosis: Comprehensive live imaging comparison and systematic review

**DOI:** 10.1371/journal.pone.0176472

**Published:** 2017-05-09

**Authors:** Tamara Hoppenbrouwers, Anouchska S. A. Autar, Andi R. Sultan, Tsion E. Abraham, Wiggert A. van Cappellen, Adriaan B. Houtsmuller, Willem J. B. van Wamel, Heleen M. M. van Beusekom, Johan W. van Neck, Moniek P. M. de Maat

**Affiliations:** 1 Department of Plastic and Reconstructive Surgery, Erasmus MC, Rotterdam, The Netherlands; 2 Department of Hematology, Erasmus MC, Rotterdam, The Netherlands; 3 Department of Cardiology, Erasmus MC, Rotterdam, The Netherlands; 4 Department of Microbiology and Infectious Diseases, Erasmus MC, Rotterdam, The Netherlands; 5 Optical Imaging Center, Department of Pathology, Erasmus MC, Rotterdam, The Netherlands; Hospital for Sick Children, CANADA

## Abstract

**Background:**

Multiple inducers of *in vitro* Neutrophil Extracellular Trap (NET) formation (NETosis) have been described. Since there is much variation in study design and results, our aim was to create a systematic review of NETosis inducers and perform a standardized *in vitro* study of NETosis inducers important in (cardiac) wound healing.

**Methods:**

*In vitro* NETosis was studied by incubating neutrophils with PMA, living and dead bacteria (*S*. *aureus* and *E*. *coli*), LPS, (activated) platelets (supernatant), glucose and calcium ionophore Ionomycin using 3-hour periods of time-lapse confocal imaging.

**Results:**

PMA is a consistent and potent inducer of NETosis. Ionomycin also consistently resulted in extrusion of DNA, albeit with a process that differs from the NETosis process induced by PMA. In our standardized experiments, living bacteria were also potent inducers of NETosis, but dead bacteria, LPS, (activated) platelets (supernatant) and glucose did not induce NETosis.

**Conclusion:**

Our systematic review confirms that there is much variation in study design and results of NETosis induction. Our experimental results confirm that under standardized conditions, PMA, living bacteria and Ionomycin all strongly induce NETosis, but real-time confocal imaging reveal different courses of events.

## Introduction

Neutrophil extracellular traps (NETs) formation, also called NETosis, is considered one of the defense mechanisms against pathogens [1]. During NETosis, the nucleus of a neutrophil decondenses and the nuclear envelope breaks, mixing chromatin, cytoplasmic and granular components. Also, the cell membrane breaks, followed by an extrusion of the neutrophil’s DNA, histones and antimicrobial proteins into the extracellular space [1]. Subsequently, pathogens are trapped in the NETs and either killed by the toxicity of the antimicrobial substances of the NETs, or immobilized to facilitate phagocytosis by other neutrophils or macrophages [2]. NETs have been shown to play a role in multiple diseases, such as thrombosis [3–6], fibrotic diseases [7], cardiovascular diseases [8] and sepsis [9–11]. Therefore, elucidation of the mechanism behind NETosis has become an increasingly important topic.

Many stimuli have been reported to induce NETosis [12]. Gram positive [1, 13, 14] and negative bacteria [1, 14] and fungi [15] induce NETosis and subsequently are trapped in the NETs. Many other inducers have also been described, but their NETosis inducing capabilities are not consistent. These include lipopolysaccharides (LPS) [1, 10, 16–18], inflammatory cytokines such as IL-6 [19] and IL-8 [1, 16, 18, 20] and the calcium (Ca^2+^) ionophore A23187 and Ionomycin [21, 22]. A difference in experimental methods and definitions of NETosis might contribute to these conflicting results.

For *in vitro* studies, phorbol 12-myristate 13-acetate (PMA), a plant derived organic compound and well-known activator of the ubiquitous signal transduction enzyme protein kinase C (PKC), is often used as an inducer of NETosis [1, 3, 12, 18]. So even though PMA is consistently reported as NETosis inducer, it is not physiologically relevant, since it does not activate physiological processes *in vivo*. Therefore, it is important to study the effects of other, physiological, NETosis inducers. This is especially relevant in studies on human diseases, such as cardiovascular wound healing, in which NETs also play a role. Results from published studies often cannot be compared because they are derived from a multitude of experimental settings. Therefore, we first made a comprehensive systematic review to make an overview of inducers of interest in cardiovascular wound healing. Subsequently, we selected the most relevant inducers and tested their effect on NETosis induction in a standardized experimental setup using static conditions and imaged using time-lapse confocal imaging.

## Material and methods

### Systematic literature review

A systematic literature review of the Medline-Ovid, Embase, Web of Science and Cochrane databases was conducted, using search and selection criteria according to the PRISMA-2015 criteria for writing a systematic literature review [23] (S2 File). MeSH-terms for “neutrophil extracellular traps” were not available. We therefore used the search terms “neutrophil extracellular traps” and/or “NET(osis)”. Moreover, we included only journal articles about *in vitro* NET induction, and only inducers that were described as inducer of NETosis by at least two papers. Only journal articles of which the full-text was available to us were included. Reviews were excluded. MEDLINE-OVID: (neutrophil extracellular trap* [TIAB] OR NETosis [TIAB]) NOT Review NOT patient* [TI] Select “journal article”. Articles were included up to January 2017. This review was executed independently by two researchers to prevent bias. Outcomes of this review were used for creating an overview to perform comparison experiments only.

### Neutrophil isolation

Neutrophils were isolated from blood from healthy donors using density gradient medium Lymphoprep^TM^ (Stem cell Technologies, Grenoble, France). All experiments were approved by the Medical Ethics Committee of the Erasmus MC. Blood was diluted 1:1 with PBS (Phosphate Buffered Saline without Ca^2+^/Mg, 17-516F, Lonza, Walkersville USA), loaded onto the Lymphoprep^TM^ and centrifuged at 830 x g for 15 minutes at room temperature. Erythrocytes were lysed by incubation with erythrolysis buffer (3.1M NH_4_Cl, 0.2M KHCO_3_, 0.02M EDTA, pH 7.4) for 10 minutes at room temperature followed by centrifugation at 690 x g for 8 minutes at room temperature. Cells were washed two times (690 x g for 8 minutes and 560 x g for 5 minutes) with HEPES buffer (0.115M NaCl, 0.012mM CaCl_2_, 1.51mM MgCl_2_, 4mM KCl, 0.01M HEPES, pH 7.4) and the concentration of cells was determined using a ABX Micros 80 cell counter (Horiba, Irvine, California).

Neutrophils were transferred to DMEM culture medium containing 10% FCS, L-glutamine and Penicillin/Streptomycin (all from Biowhittaker, Lonza, Walkersville, USA) or DMEM culture medium without any additions for bacterial experiments. Hoechst 34580 (1:10 000, Life Technologies, Landsmeer, The Netherlands) for staining DNA and Propidium Iodide (PI, 1:400, Sigma Aldrich, Zwijndrecht, The Netherlands) for staining extracellular DNA were added and cells were incubated for at least 1 hour at 37°C on gelatin-coated (Sigma Aldrich, Zwijndrecht, The Netherlands) 24 wells glass-bottom plates.

### Selection of NETosis inducers for *in vitro* experiments

From the inducers we documented in our search we selected inducers that were well described and play a role in (cardiovascular) wound healing. We then selected the best described inducers as well as the inducers with the most variation in reported effect to test in our own standardized experimental setup. As a source for cytokines, we used activated platelets supernatant, containing cytokines such as IL-8, PDGF and VEGF [24, 25].

### Bacterial strains and culture

Gram-positive (*S*. *aureus* Newman) and gram-negative (*E*. *coli* ATCC 25922 (O6:B1)) bacteria were cultured in 100 ml Iscove’s Modified Dulbecco’s Media (Gibco® IMDM medium, Life Technologies, Landsmeer, The Netherlands) at 37°C overnight. The next day the bacteria were diluted to a final concentration of 10^8^ bacteria per ml as determined by OD_600_ measurements. For experiments with dead bacteria, the bacteria were either killed by incubation at 90°C for 10 minutes or by exposure to UV light with 6000 μWs/cm^2^ for 66 seconds.

### NETosis induction and time-lapse imaging

Isolated neutrophils (10^7^ cells/well) were added to a 24 wells plate in a final volume of 500 μl. Stock solutions of PMA and Ionomycin were prepared in dimethyl sulfoxide (DMSO, Sigma Aldrich). Platelets (platelet rich plasma) were isolated from EDTA blood by centrifugation for 7 minutes at 260 x g without brake, and activated for 10 minutes by adding thrombin (1 U/ml). Activated platelets supernatant was collected by centrifuging the activated platelets at 2000 x g for 10 minutes.

To induce NETosis, non-bacterial inducers were individually added to each well. Before addition (t = 0), an image was taken and starting directly after addition of the inducers, cells in a random field were imaged every 15 minutes for 3 hours with a 20x 0.7 n.a. lens by using confocal microscopy (Leica SP5 AOBS, Leica Microsystems, Wetzlar, Germany). Excitation with a 405 laser and a BP 420–500 emission filter for Hoechst and a 561 excitation and BP 580–620 emission filter for PI. In this setting, the dish was mechanically moved between fields. We stopped imaging after three hours, since after three hours spontaneous cell death was observed in control neutrophils. In experiments containing bacteria, we imaged continuously for one hour since all neutrophils underwent NETosis within one hour in all bacterial conditions. We defined NETosis as a host defense mechanism in which neutrophils release their nuclear and granular contents to contain and kill pathogens. The NETs that are released form extensive webs of DNA coated with cytotoxic histones and microbicidal proteases. In cells that stained only positive for Hoechst, the cell membrane was still intact. After breakdown of the cell membrane, the DNA became PI positive. Unstimulated cells (in experiments without platelets) and resting platelets were used as negative control and PMA stimulated cells were used as positive control.

### Immunofluorescence

To confirm *in vitro* NETosis in the bacteria experiments, we added an immunofluorescent staining with a MPO-Dylight488 complex (1:250) to the neutrophils immediately before induction. Then, we quantified the positive NETs by using confocal microscopy (Leica SP5 AOBS).

As another measurement for NETosis, cells were stimulated by the described inducers for 3 hours, fixed and stained for myeloperoxidase (MPO, Dako). Briefly, after antigen retrieval with Proteinase K, the slides were blocked with skim milk powder (5%) in PBS Tween 0.1% pH7.4 and incubated overnight with polyclonal rabbit anti-human MPO (1:300) at 4°C. After washing with PBS Tween 0.1% pH7.4 slides were incubated with secondary antibody Dylight goat anti rabbit 488 (1:200) for 30 minutes. Slides were mounted with Prolong Diamond antifade with DAPI (Thermofischer). Images were made by using confocal microscopy (Leica SP5 AOBS) and Structured Illumination Microscopy (Zeiss Elyra PS1 LSM 780 structured illumination microscope, Carl Zeiss, Jena, Germany).

### Image analysis

All images were analyzed using ImageJ (Version 1.49, National Institutes of Health, USA). We quantified the number, area and mean intensity of Hoechst positive and PI positive cells using a macro that includes a segmentation of the nuclei on a Gaussian blurred image (sigma = 2px) with a threshold and a watershed segmentation (S3 File). A minimal and maximal size of Hoechst positive and PI positive cells was included in the macro. The Hoechst and PI threshold was kept constant within one experiment. We determined the ratio of PI positive cells and corrected at t = 0 for dead cells in the start mixture. To correct for regular cell death during the experiment, conditions were compared to the negative controls: no additions or resting platelets, in which no NETosis was observed.

### Statistics

All data are presented as mean±SEM. A repeated measurements ANOVA was used to detect differences in NET ratio with time and inducer as independent parameters. Results were considered statistically significant when p<0.05. Data were analyzed using SPSS v22 (IBM, USA).

## Results

### Systematic literature review

**Fig 1:**
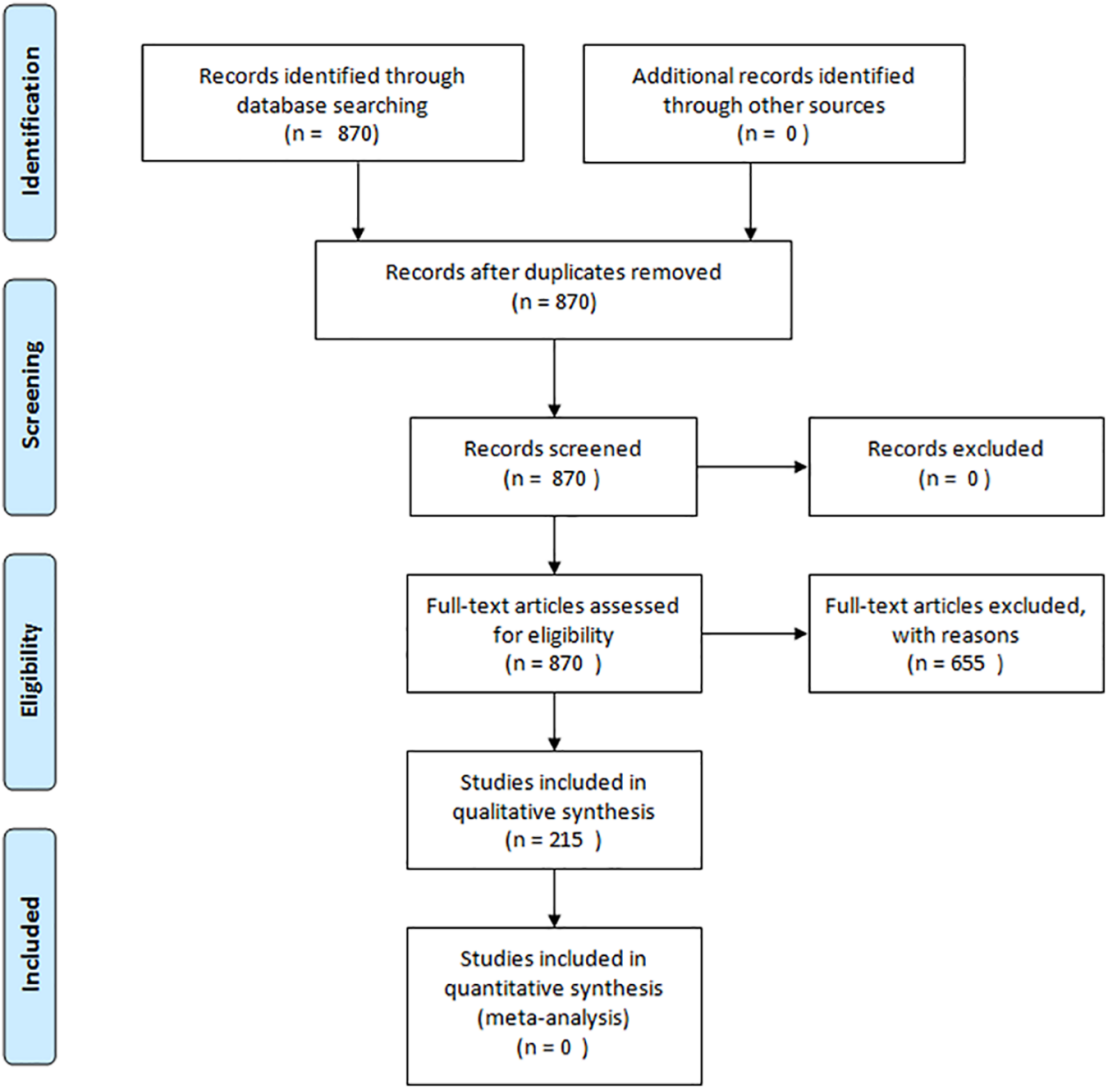
PRISM chart of the systematic literature review.

Our systematic search strategy resulted in 870 scientific articles, of which 655 were excluded following selection according to the described criteria (Fig 1). In the 215 remaining articles we identified 25 different NETosis inducers. These inducers are presented in Table 1.

**Table 1:** Overview of in-literature described *in vitro* NETs inducers. MOI: Multiplicity Of Infection (number of bacteria to number of cells). CFU: Colony Forming Units.

Inducer	Concentration	Induction time	NETosis	Reference
PMA	4–50 nM (3–30.8 ng/ml)	10 min-16h	Yes	[1–3, 12, 16, 19, 20, 26–112]
	60–100 nM (37–62 ng/ml)	30 min-16h	Yes	[7, 18, 21, 113–146]
	120–1620 nM (74–1000 ng/ml)	10 min-4h	Yes	[147–166]
	100000 nM (6168 ng/ml)	10 min-24h	Yes	[14, 167, 168]
H_2_O_2_	0.1 μM	3h	No	[116]
	100–1000 μM	4h	Yes	[61, 114]
	4000 μM	200 min	Apoptosis	[18]
	10000 μM	200 min	Necrosis	[18]
	10000 μM	4-5h	Yes	[54]
	0.03%	3h	Yes	[169]
**Growth factors/platelets**				
IL-8	1–250 ng/ml	10 min-5h	Yes	[1, 12, 27, 29, 42, 64, 161, 170–172]
	10 ng/ml	3h	Little	[16]
	100–800 ng/ml	4-18h	No	[18, 76, 82]
IL-1β	10 ng/ml	6h	Little	[62]
	50 ng/ml	2h	Yes	[27]
TNF-α	1 ng/ml	6h	Little	[62]
	7–20 ng/ml	30 min-5h	Yes	[19, 20, 54]
	100 ng/ml	2h	Yes	[27]
	100 ng/ml	4h	No	[76]
Platelets	5x10^7^ /ml		No	[10]
	2x10^5 –^ 5x10^5^	1h	No	[44]
Activated platelets	2x10^5 –^ 5x10^5^ (+ 50 μM TRAP)	1h	Yes	[44]
	5x10^5^ (+ 1.3 μg/mL collagen)	2h	Yes	[173]
	1:400 (+ 0.01 U/mL Thrombin)	4h	Yes	[174]
	25–100 ml (+ 5 μmol/L PGE1)	20 min	Yes	[175]
	25–100 ml (+ 25 μmol/l TRAP-6)	20 min	Yes	[175]
	25–100 ml (+ 5 μmol/l ADP)	20 min	Yes	[175]
	25–100 ml (+ 1 μg/ml collagen)	20 min	Yes	[175]
	25–100 ml (+ 0.05 IU/mlrecombinant thrombin)	20 min	Yes	[175]
	(+CoCr)		Yes	[176]
**Calcium**				
A23187	0.2–25 μM	20 min-4h	Yes	[22, 130, 151, 177–179]
	1 μM	1h	No	[180]
	100 μM	1-4h	Little	[167]
Ionomycin	0.9–7 μM	30 min-4h	Yes	[21, 29, 34, 130, 132]
	100 μM	1-4h	Little	[167]
MSU crystals	100–200 μg/ml	3-5h	Yes	[71, 118, 181]
	1000 μg/ml	2h	Yes	[182]
			Yes	[134]
	20 pg/cell	2h	Yes	[80]
**Glucose**				
Glucose Oxidase	100 mU/ml	1-4h	Yes	[12, 172]
Glucose	5.5–10 nM	2h	No	[183]
	20–30 nM	2h	Yes	[183]
	5000000 nM	3h	No	[58]
	25000000 nM	3h	Yes	[58]
**Bacterial/fungal products**				
LPS	0.1 ng/ml	1h	Yes	[184]
	0.1–10 μg/ml	15 min-18h	Yes	[1, 19, 28, 54, 61, 64, 71, 76, 82, 83, 147, 158, 161, 185–194]
	0.1–25 μg/ml	2,5-3h	Little	[16, 21]
	0.3–5 μg/ml	15 min	No	[10, 17]
	50 μg/ml	30–90 min	Yes	[155, 195]
			No	[18]
	10 mg/L	30 min	Yes	[190]
		30 min	Yes	[196]
LPS + Glucose	2 μg/ml + 30000000 nM	3h	Little	[19]
	2.5–25 μg/ml	2,5h	Yes	[21]
LPS + Platelets	1–5 μg/ml + 5x10^7^-2.4x10^8^ /ml	30 min +	Yes	[10, 197]
	25–100 ml +	20 min	Yes	[175]
β-glucan	200 μg/ml	15–240 min	Yes	[198, 199]
	1000 μg/ml	1h	Yes	[129]
**Bacteria/fungi**				
*S*. *aureus*	0.03–50 MOI	30 min-24h	Yes	[12, 14, 34, 40, 71, 116, 169, 200–202]
	6x10^6^/ml	1h	Yes	[180]
	25 μl OD 0.5	3h	Yes	[86]
*S*. *pneumonia*	10 MOI	10 min-24h	Yes	[14]
*S*. *pneumonia* (dead)	2x10^7^/ml	4h	Yes	[118]
*E*. *coli*		4h	No	[15]
	3–50 MOI	10 min-24h	Yes	[14, 34, 79, 124, 202]
	100 MOI	1-4h	Yes	[51, 184]
	10^6–^10^7^ CFU	5min-1h	Yes	[189, 203]
	2000 CFU	1-8h	Yes	[189]
*P*. *aeruginosa*	1–50 MOI	5h	Some	[204]
	10–100 MOI	10 min-24h	Yes	[14, 34, 168, 205–207]
		8h	Yes	[208]
	6x10^6^/ml	1h	Yes	[180]
*A*. *fumigatus* (hyphae)	750 CFU / 50 μl	2h	Yes	[209]
	0.2–2000 MOI	40–180 min	Yes	[64, 77]
	10^6^ conidia	3h	Yes	[191]
*C*. *albicans* (yeast)	0.5 MOI	90 min	Little	[125]
	2 MOI	15 min	Yes	[210]
	2 MOI	4h	No	[210]
	5 MOI	3h	No	[148]
	10 MOI	3h	Little	[120]
	10 MOI	2h	Yes	[123]
*C*. *albicans* (hyphae)	0.2–4.2 MOI	5-min–4h	Yes	[125, 126, 210–212]
		30 min	Yes	[213]
*M*. *bovis*	10 MOI	4h	Yes	[177]
	10–1000 MOI	1-4h	No	[159]

PMA is the most frequently used stimulus with a 100% success rate for inducing NETosis. In literature different concentrations are used ranging from 5 nM to 100 μM. NETosis was observed within a time frame ranging from 10 minutes to 24 hours [1–3, 7, 12, 14, 16, 18–21, 26–112, 113–146, 147–168].

*S*. *aureus* has consistently been described to be a potent inducer of NETosis [12,14,34,40,71,75,104,131–133]. In literature search a variety of other bacteria were reported, that also induced NETosis, although most species are weak NETosis inducers compared to *S*. *aureus* [14]. *E*. *coli P*. *aeruginosa*, *C*. *albicans* yeast and *M*. *bovis* have also been described as potent NETosis inducers in most papers, but discrepancies occur [15, 148, 159, 207, 210].

The NETosis inducing properties of LPS have been investigated in many papers, but the results are contradicting. For example, in LPS-activated neutrophils multiple papers state to have observed NETosis after 30 min with 100 ng/ml [76, 83, 158, 161, 187], whereas other authors did not observe NETosis using a concentration of 10 μg/ml [18].

Results for glucose as an inducer for NETosis indicate that higher concentrations (20–30 mM) of glucose appear to induce NETosis whilst low concentrations (5–10 mM) do not [58, 187]. Higher concentrations of glucose are thought to resemble a hyperglycemic environment for neutrophils and may mimic the situation in patients with badly regulated Diabetes Mellitus. NETosis induced by glucose therefore seems concentration dependent.

Studies with A23187 report conflicting results. Six studies reported induction of NETosis after stimulation with 5μg/mL and 0.2–25 μM for 20 minutes to 4 hours [22, 130, 151, 179, 181, 183]. Two other studies reported little to no NETosis after induction with 1 and 100 μM of A23187for 1–4 hours [167, 182]. Ionomycin is also reported to induce NETosis after 30–180 min [21, 29, 34, 130, 132, 167, 195].

Experiments with IL-8 as an inducer of NETosis gave various results. In one study, NETosis was induced between 30–240 min after administration of 10-100ng/ml IL-8 [12]. However, in other studies, after stimulation with 200-800ng/ml IL-8 for 4-18h NETosis was not observed [18, 76, 82]. TNF-α is reported as an inducer of NETosis in five studies, while two papers report little or no NETosis. NETosis was observed 30 minutes to 6 hours after administration of 7-100ng/ml TNF-α [19, 20, 27, 54, 62]. One study used a concentration of 1ng/mL and reported little effect of TNF-α as an inducer, and one study did not observe NETosis at all after 4h with 100ng/ml [76].

Another investigated inducer was H_2_O_2_. Some experiments including H_2_O_2_ did not show clear NETosis but showed other forms of cell death such as apoptosis and necrosis [18, 116]. However, H_2_O_2_ also was reported, by other studies, to be a good inducer of NETosis [54, 61, 114, 169].

In summary, the data in literature show that PMA is a well-defined inducer of NETosis with a 100% success rate. Bacterial inducers of NETosis such as *S*. *aureus* (10:1–20:1 bacteria to neutrophils) also seem consistent inducers, but in some strains discrepancies occur and the process is less well described than PMA. Other inducers, such as cytokines IL-8 and activated platelets, different glucose concentrations and especially LPS, display a variable outcome.

Our literature search revealed the observation that numerous experiments have been performed in which it became clear that all inducers, with the exception of PMA, have been studied with experimental conditions that differed between studies, such as time frame, concentration and NETs imaging procedure. This could partly explain the observed differences in NETosis induction.Hence, there is a need for a well-controlled evaluation of NETosis inducers. We therefore performed a standardized study in which we tested the NETosis capability of different NETs inducers (as defined in Table 2). Bacterial infections, diabetes and calcium influx all influence cardiovascular wound healing differently. Therefore, we selected *S*. *aureus*, *E*. *coli*, LPS, Ionomycin, glucose and combinations with (activated) platelets and LPS for our panel. PMA will be taken as a positive control, whilst unstimulated cells are a negative control in experiments without platelets, and resting platelets are a negative control in experiments with platelets. In this study, we use a well-defined experimental setup to test multiple conditions at the same time on the same neutrophils.

**Table 2:** Concentrations of the potential NETosis inducers in the experiments.

NETosis inducer and final concentrations
PMA (Sigma Aldrich, Saint Louis, Missouri, USA)
• 50 ng/ml • 250 ng/ml
Platelets (isolated from EDTA blood)
• 5x10^7^ /ml
Supernatant of activated platelets (isolated from EDTA blood)
• 5x10^7^ /ml
D-Glucose (Amresco)
• 25 μM • 25 mM
Ionomycin (Sigma Aldrich)
• 3 μg/ml • 5 μg/ml
LPS (Sigma Aldrich): source
*E*. *coli* O55:B5
• 10 ng/ml • 100 ng/ml • 1000 ng/ml • 5 μg/ml
*E*. *coli* O111:B4
• 10 ng/ml • 100 ng/ml • 1000 ng/ml • 5 μg/ml
*P*. *aeruginosa*
• 10 ng/ml • 100 ng/ml • 1000 ng/ml • 5 μg/ml
Platelets + LPS (*E*. *coli* O111:B4)
• 5x10^7^ /ml + 5 μg/ml
Activated platelets supernatant + LPS (*E*. *coli* O111:B4)
5x10^7^ /ml + 5 μg/ml
Living bacteria
*S*. *aureus* (Newman)
• 10^8^/ml (±10:1)
*E*. *coli* ATCC 25922 (O6:B1)
• 10^8^/ml (±10:1)
Dead bacteria
*S*. *aureus* (Newman)
• 10^10^/ml (±1000:1)
*E*. *coli* ATCC 25922 (O6:B1)
• 10^10^/ml (±1000:1)

### NETosis experiments

#### PMA

**Fig 2:**
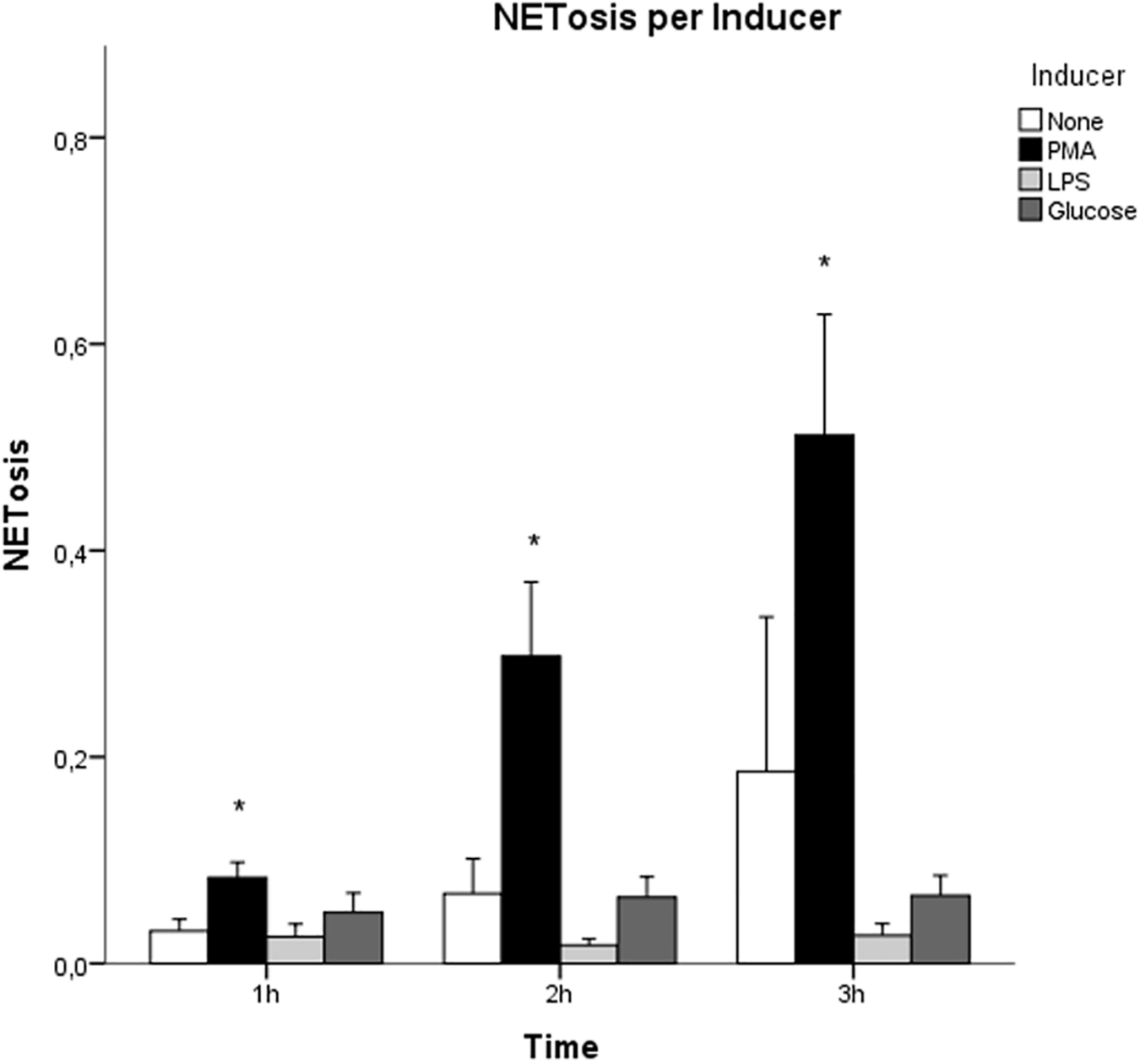
NETosis induction for the different inducers. NETosis was defined as the ratio between the number of Hoechst and PI positive cells. PMA induced NETosis when compared to unstimulated neutrophils, p<0.001 repeated measures ANOVA post-hoc Bonferroni (*) (none n = 5, PMA n = 7, LPS n = 7, glucose n = 5). Error Bars +/- SEM.

In our experiments PMA (n = 7) consistently and strongly induced NETosis (61.5 ± 9.3% of PMA stimulated neutrophils vs 4.1 ± 1.3% of unstimulated neutrophils, p<0.001) (Table 3, Fig 2 and Fig A in S1 File). NETosis was observed about 1.5 hours after administration of PMA and observed for both concentrations (50 ng/ml and 250 ng/ml).

**Table 3:** Percentage of neutrophils that underwent NETosis. % NETosis per time point (hr) is given as mean (SEM). P-value of repeated measures ANOVA with Bonferroni post-hoc test results per NETosis inducer versus unstimulated neutrophils.

	n	Time (hr)				p-value
		0	1	2	3	
*None*	5	1.94 (0.43)	3.8 (1.35)	3.68 (1.08)	4.10 (1.34)	n.s.
*PMA* (250 ng/ml)	7	3.89 (0.91)	7.81(1.64)	31.94 (6.17)	61.52 (9.34)	<0.001
*LPS* (5 μg/ml)	7	2.98 (1.58)	2.62 (1.26)	2.90 (1.08)	3.98 (1.69)	n.s.
*Glucose* (25 mM)	3	4.71(1.06)	4.92 (1.90)	6.40 (1.98)	6.58 (1.96)	n.s.
*Platelets* (5x10^7^)	5	2.53 (0.71)	2.80 (1.12)	1.12 (0.89)	1.16 (0.92)	n.s.
*Activated Platelets* (5x10^7^)	7	2.00 (0.60)	3.24 (1.15)	1.45 (0.61)	1.27 (0.47)	n.s.
*Activated Platelets Supernatant* (5x10^7^)	7	2.84 (1.12)	4.48 (1.19)	5.68 (2.31)	5.94 (2.45)	n.s.
*Platelets and LPS* (5x10^7^ + 5 μg/ml)	7	2.60 (0.79)	3.57 (0.72)	2.43 (1.09)	3.09 (0.98)	n.s.
*Activated Platelets and LPS* (5x10^7^ + 5 μg/ml)	7	2.35 (0.77)	2.75 (1.34)	2.65 (0.93)	3.77 (1.09)	n.s.
*Activated Platelets Supernatant and LPS* (5x10^7^ + 5 μg/ml)	7	2.87 (1.10)	6.86 (1.84)	7.01 (2.88)	7.88 (3.56)	n.s.

#### Living bacteria

**Fig 3:**
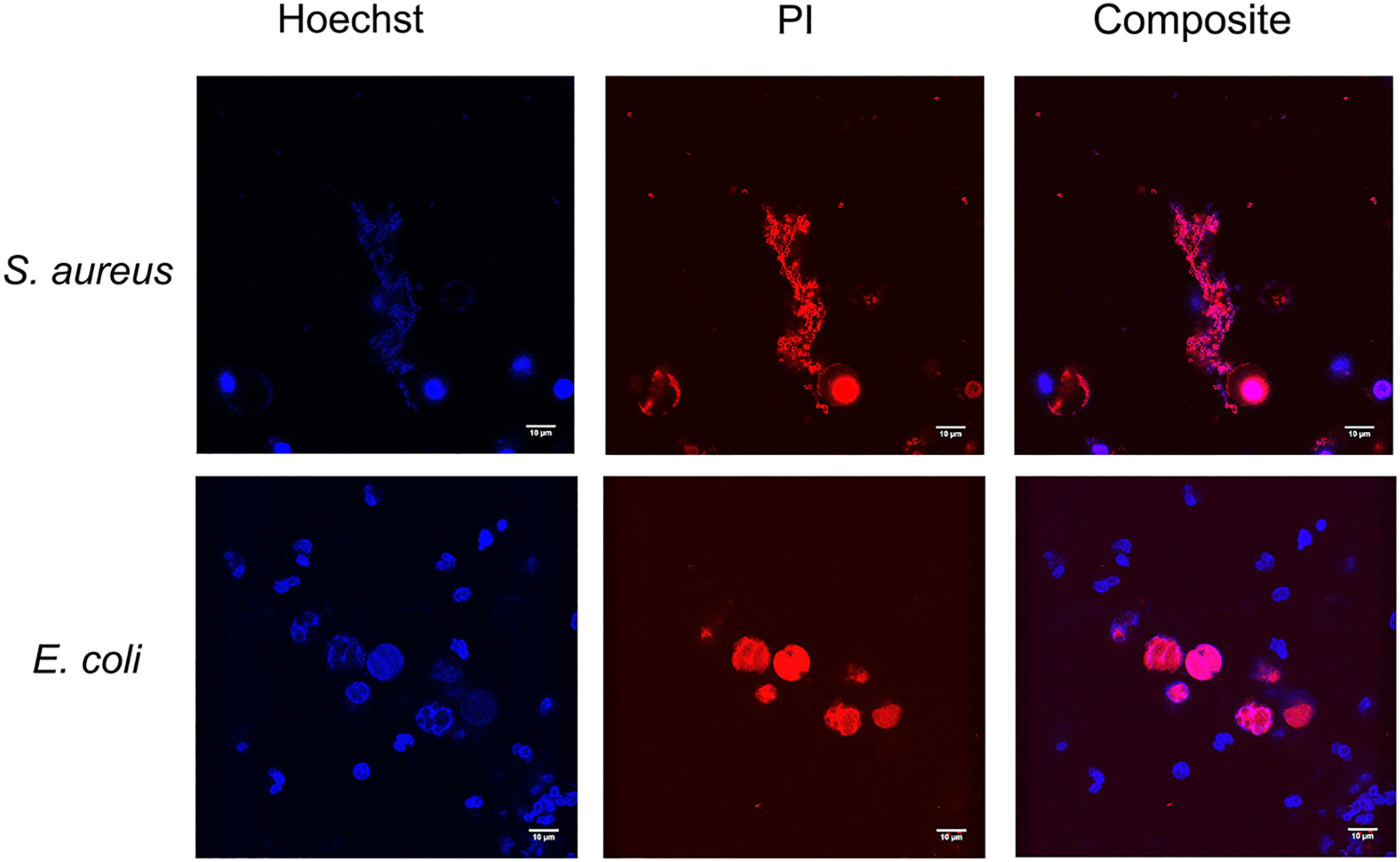
NETs formed by *S*. *aureus* and *E*. *coli* 20 minutes after stimulation for one hour. DNA (Hoechst, blue, 405) and Extracelullar DNA (PI, red, 561) were stained.

In our experiments both gram positive and gram negative bacteria strongly induced NETosis. In *S*. *aureus* stimulated samples (n = 3), NETs were observed after 10–20 minutes and in *E*. *coli* stimulated samples (n = 3), NETs were observed within one hour (Fig 3), as confirmed by live MPO staining (Fig B in S1 File). NETs induction by both bacteria strains differed in the amount of viable (Hoechst positive) neutrophils. After the addition of *S*. *aureus*, no Hoechst positive neutrophils were observed after 40 minutes. After the addition of *E*. *coli*, neutrophils remained viable during the total experiment. After the addition of dead *S*. *aureus* and *E*. *coli* (n = 3), phagocytosis of the bacteria by the neutrophils and no NETosis was observed (Fig C in S1 File).

#### LPS and glucose

**Fig 4:**
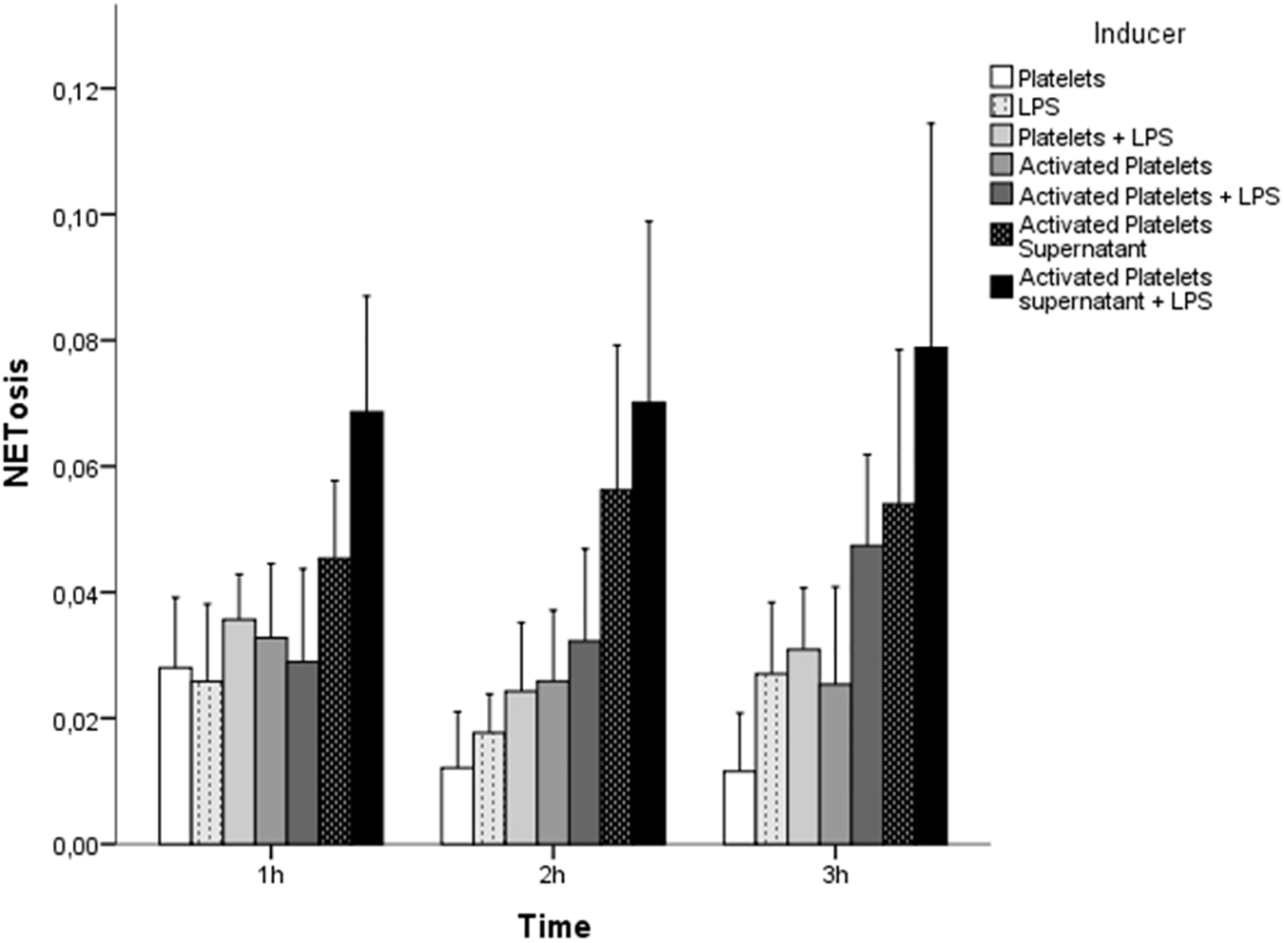
NETosis per inducer comparing the effect of platelets and the effect of activated platelets supernatant (n = 5, n = 7 respectively). NETosis was defined as the ratio between Hoechst and PI positive cells. Neutrophils stimulated by platelets were compared against neutrophils stimulated by LPS, platelets + LPS, activated platelets, activated platelets + LPS, activated platelets supernatant, activated platelets supernatant + LPS using repeated measures ANOVA post-hoc Bonferroni (*). No significant differences were found (p>0.05 all). Error Bars +/- SEM.

No NETosis was observed when neutrophils were incubated with LPS (n = 7) or glucose (n = 5). For LPS, multiple concentrations and variants (Table 2) were tested, but none induced NETosis. Also, combinations of LPS with platelets, activated platelets and activated platelets supernatant were unsuccessful in inducing NETosis (Fig 4) (n = 7 for all).

#### Ionomycin

**Fig 5:**
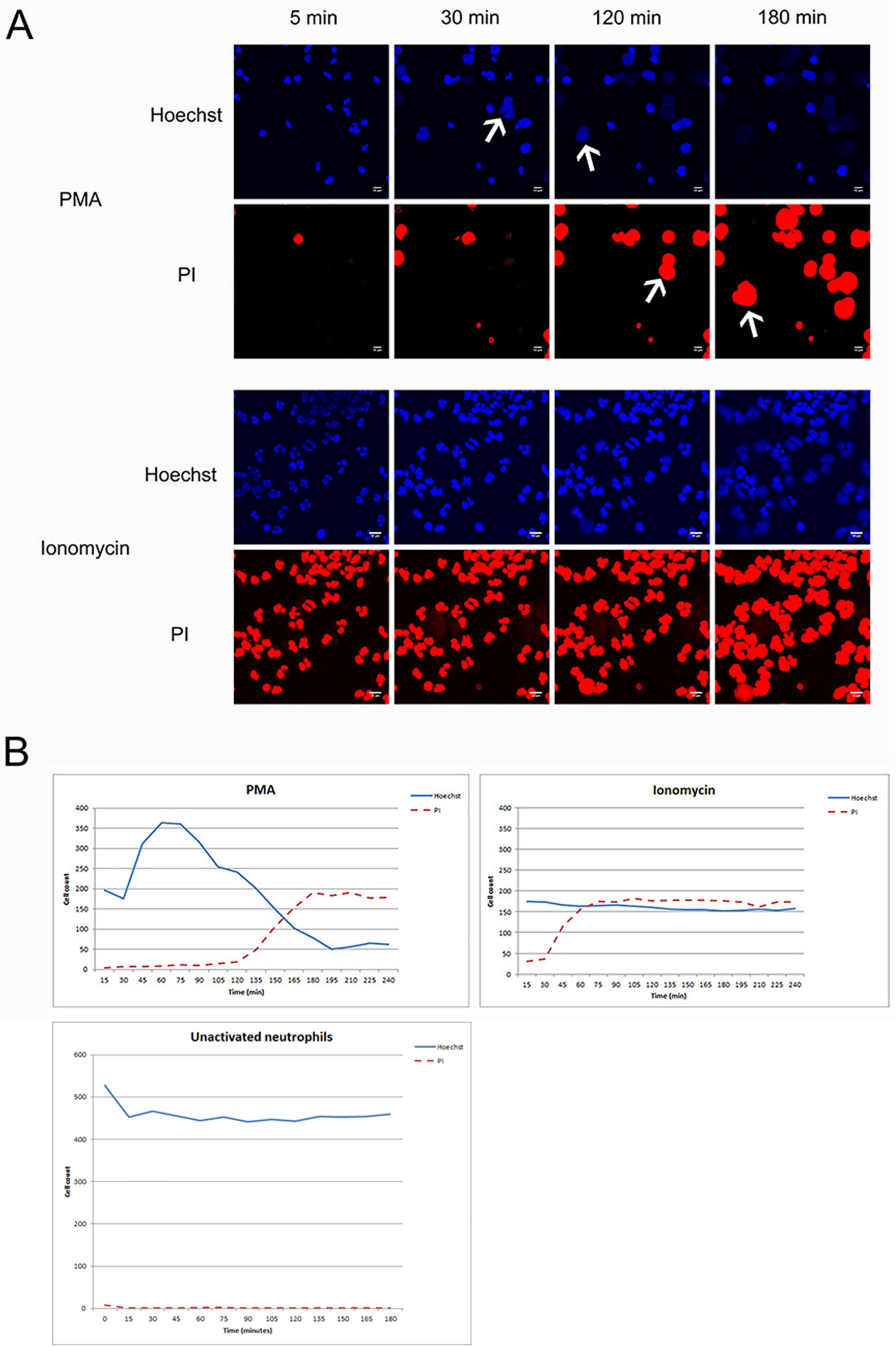
The effect of Ionomycin compared to PMA. (A) Time lapse images of PMA and Ionomycin at different time frames. The arrows indicate the decondensation of the nuclei before (Hoechst, 405) and after (PI, 561) DNA extrusion. (B) The amount of Hoechst and PI positive cells in PMA and Ionomycin stimulated cells and unstimulated cells, show the difference in the process of NETosis. In the PMA stimulated cells, the number of Hoechst positive cells go down as the PI positive cells (NETosis) go up. In the Ionomycin stimulated cells, the number of PI positive cells go up very rapidly, but the Hoechst positive cells remain similar. In unstimulated cells, the intensity of Hoechst staining remains high and no PI staining was detected.

When neutrophils were incubated with Ionomycin (n = 3), the sequence of events leading to DNA extrusion was different from the process we observed after the addition of PMA. Within 15 minutes after the addition of Ionomycin, the membranes of the neutrophils became porous, as shown by staining the nuclei for PI (Fig 5, S1 Video and S2 Video). In our three hour imaging timeframe, DNA was seen to slowly leak out of the cells. This process was not observed in cells that died as a result of necrosis or apoptosis, where the DNA remains within the cells. At the end of the experiment, the neutrophils treated with Ionomycin and the neutrophils that were incubated with PMA looked similar, and, therefore, this difference in the DNA extrusion process may be missed in studies that did not study early time points.

#### Activated platelets

We did not observe NETosis after incubating the neutrophils with thrombin activated platelets, activated platelets supernatant, platelets and LPS and activated platelets plus LPS (all n.s. n = 7). However, in two experiments, NETosis was observed after incubating the neutrophils with LPS and activated platelets supernatant, while in the other experiments (n = 7) no NETosis was induced. A possible explanation for this variation could be the variance between donors, though blood samples were taken from healthy donors, and none of the observed results could be linked to either sex or age.

## Discussion

Our *in vitro* study, performed in a well-defined and well-controlled time-lapse setting, revealed that PMA, bacteria and Ionomycin were robust inducers of NETosis. The other reported NETosis inducers were less potent.

First, we performed a systematic literature review of NETosis inducers. This is the first systematic review to address NETosis inducers. NETosis is currently intensively investigated and therefore a systematic review on this topic is very needed. Our literature search revealed that PMA and bacteria were consistent inducers of NETosis. Both are being used in a more routine way in research now. PMA is used to mainly investigate the effect of other inducers and the ROS-pathway.

Studies on other inducers presented conflicting results. The difference in experimental setting, timing and dosing might contribute to the variation in results. Therefore, we performed *in vitro* experiments in a standardized laboratory setting. In these experiments, we used concentrations based on literature and a time frame of 3 hours, the period in which the neutrophils remained viable. Our experiments confirmed the robustness of NETosis inducers PMA and bacteria. PMA was also used as a positive control in our experiments, as it was a consistent inducer throughout the literature. In the imaging of NETosis by Ionomycin, we observed a different sequence of events, but according to the definition of NETosis that we use in this paper, Ionomycin is also a qualified inducer. NETosis was not observed with other tested inducers.

### Bacteria and bacterial products

In our standardized experiments, living gram negative as well as living gram positive bacteria were strong and consistent NETosis inducers. Several studies, using a variation of experimental conditions, support our findings [12–15, 214].

Our study also showed that different species gave a different time of onset of NETosis and a different percentage of the neutrophils that underwent NETosis. This is in line with Pilsczek *et al* [14]. We saw more NETosis after the induction with *S*. *aureus* compared to *E*. *coli*.

Dead bacteria did not induce NETosis in our experiments. Isolated LTA (derived from gram-positive bacteria) and LPS (derived from gram-negative bacteria), both bacterial-wall proteins, have been described to induce NETosis. We hypothesized that dead bacteria also expose these proteins and, therefore, were expected to be potent NETosis inducers. We killed the bacteria with two methods (heat and UV), however, did not observe NETosis in either situation.

When we added LPS, no NETosis was induced. In literature, contradictory reports are found regarding LPS as a NETosis inducer. To test whether the type of LPS explains this contradiction, we used three different types of LPS (derived from *E*. *coli* O55:B5, *E*. *coli* O111:B4 and *P*. aeruginosa), which also were used in literature. Data are shown in Fig 2. Post Hoc testing showed no difference between LPS and unstimulated neutrophils. These results are in line with our experiment where dead gram-negative bacteria, with LPS on the surface, also failed to induce NETosis. Therefore, the ability of LPS to induce NETosis should be studied further.

### Glucose

In literature, glucose is described as NETosis inducer [58, 183]. In our experiments, glucose did not induce NETosis. One study suggested that high levels of glucose make neutrophils more sensitive to NETosis inducers such as cytokines or LPS [58]. In contrast, other literature that claims that neutrophils become insensitive to stimuli when maintained in high glucose concentrations [19].

### Calcium ionophore

Incubation of the neutrophils with the Ionomycin resulted in an extrusion of DNA, however, this process differed from the PMA induced NETosis. Ionomycin opens the calcium channels of cells, thus causing high intracellular Ca^2+^ levels. This resulted in pore formation in the cellular membranes and positive staining for PI in the cells, followed by leakage of PI-positive material out of the cells. In the NETosis induced by PMA, nuclear swelling is seen as the first step. Still, we considered the process after Ionomycin induction NETosis, since in other forms of cell death, i.e. necrosis and apoptosis, the nuclear envelope remains intact, which prevents DNA excretion from the dead cell [12].

We emphasize that it is important to visualize the whole NETosis process and not only rely on end stage measurements. We have shown that there is variation in the process of DNA extrusion with Ionomycin. Since other studies on Ionomycin only measured at the end of the NETosis process, they may have missed these variations.

### Platelets

In our experiments, resting platelets did not induce NETosis. This results is in contrast with literature [10, 44]. We also did not see NETosis induction when we incubated neutrophils with activated platelets or activated platelets plus LPS, as described by one other study [175]. The majority of the studies, however, described that the excretion of growth factors by activated platelets (stimulated by for example LPS or PAF) will activate neutrophils and stimulate NETosis [10, 44, 173, 174].

### Difference in neutrophil function

Interestingly, we observed that NETosis induction with activated platelets was variable amongst healthy individuals, since strong NETosis was observed in two samples while absent in five other samples. Our donors were healthy individuals. Individual variation in neutrophil response might be an explanation for variable results. Therefore, all experiments with neutrophils should include blood samples from multiple healthy donors and should be repeated multiple times to obviate as much variation as possible.

### Study limitations and recommendations

To our knowledge this is the first *in vitro* study that compares a comprehensive panel of NETosis inducers under standardized experimental conditions using time-lapse imaging, allowing a direct quantification of the NETosis strength using image analysis to quantify the data. We consider it a strong point of our study that time-lapse images allow visualization of the actual NETosis process. Therefore, NETosis can be identified with higher certainty than when using single images. Our approach was, for example, very helpful in interpreting the experiments with Ionomycin. In our study our medium contained 10% FCS. While this is widely used for cell culture purposes, in NETosis experiments this could affect NETosis, since FCS contains nucleases, which have been described to break down NETs *in vitro* [215]. However, nucleases break down the NETs after they have formed, and we did not observe this in our time-lapse analysis. Also, it is unlikely that either the use of DMEM or FCS would have an effect on any of the tested inducers. For example, the most used medium for NETosis experiments is RPMI-1640, but studies have also reported LPS, one of the most contradictive inducers in our panel, not to have much effect in their studies [16, 18, 21]. We are aware that Ca^2+^ in buffers can have an effect on NETosis. Therefore, we used PBS free from Ca^2+^, and our HEPES buffer only contained a minimal concentration of 12 μM Ca^2+^.

A drawback of any *in vitro* setting obviously is that an *in vitro* setup cannot completely reflect the *in vivo* situation. Inducers like LPS also are expected to trigger an immune response *in vivo*, which could trigger alternate pathways that induce NETosis.

NETosis can be found in many pathological conditions such as thrombosis and sepsis, which leads to a rising interest in exploration of its pathways. These pathways could be further explored *in vitro* in a setup similar to ours.

## Conclusion

Our literature research showed that living gram positive and negative bacteria, PMA and Ionomycin are strong NETs inducers. Other inducers are less potent. Our additional experiments, which were performed under one experimental condition confirmed these our results found during our literature research.

## Supporting information

S1 File **Fig A:** Immunofluorescence shows the localization of DNA (blue, 405) and MPO (green, 488) in unstimulated (left) and PMA stimulated (right) neutrophils. **Fig B:** NETs formed by S. aureus after 20 minutes of stimulation, stained with MPO-Dylight488, PI and Hoechst. **Fig C:** Bright field overlay image of neutrophils (blue) with dead bacteria (red) showing phagocytosis.
(DOCX)

S2 File PRISMA Checklist. (PDF)

S3 File Macro used for quantification of Hoechst and PI positive cells. (IJM)

S4 File All raw data as loaded into SPSS for analyses. (XLSX)

S1 Video Time-lapse video of neutrophils activated with PMA. Blue: Hoechst (DNA). Red: PI (extracellular DNA).
(AVI)

S2 Video Time-lapse video of neutrophils activated with Ionomycin. Blue: Hoechst (DNA). Red: PI (extracellular DNA).
(AVI)
