# Supplementary material for: In vitro induction of NETosis: Comprehensive live imaging comparison and systematic review
Source: PLoS One. 2017 May 9;12(5):e0176472. doi: 10.1371/journal.pone.0176472 (PMC5423591; doi:10.1371/journal.pone.0176472)
Supplement: S1 File — Fig A: Immunofluorescence shows the localization of DNA (blue, 405) and MPO (green, 488) in unstimulated (left) and PMA stimulated (right) neutrophils. Fig B: NETs formed by S. aureus after 20 minutes of stimulation, stained with MPO-Dylight488, PI and Hoechst. Fig C: Bright field overlay image of neutrophils (blue) with dead bacteria (red) showing phagocytosis. (DOCX) [file pone.0176472.s001.docx]

**Supplemental Data**

**S1 Fig A**: Immunofluorescence shows the localization of DNA (blue, 405) and MPO (green, 488) in unstimulated (left) and PMA stimulated (right) neutrophils.


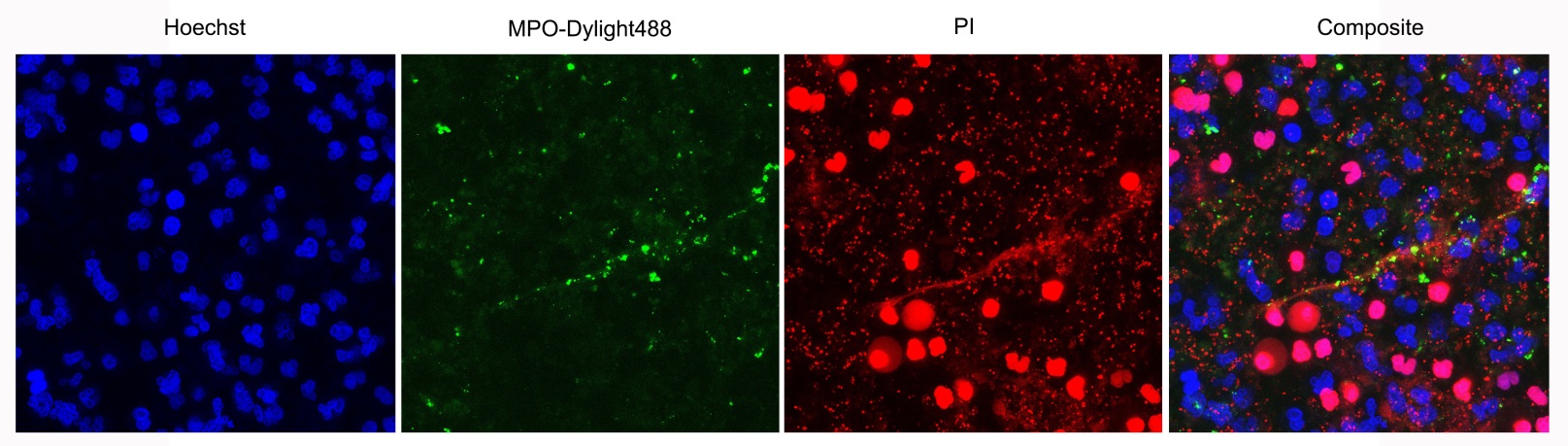


**S1 Fig B:** NETs formed by *S. aureus* after 20 minutes of stimulation, stained with MPO-Dylight488, PI and Hoechst.

*
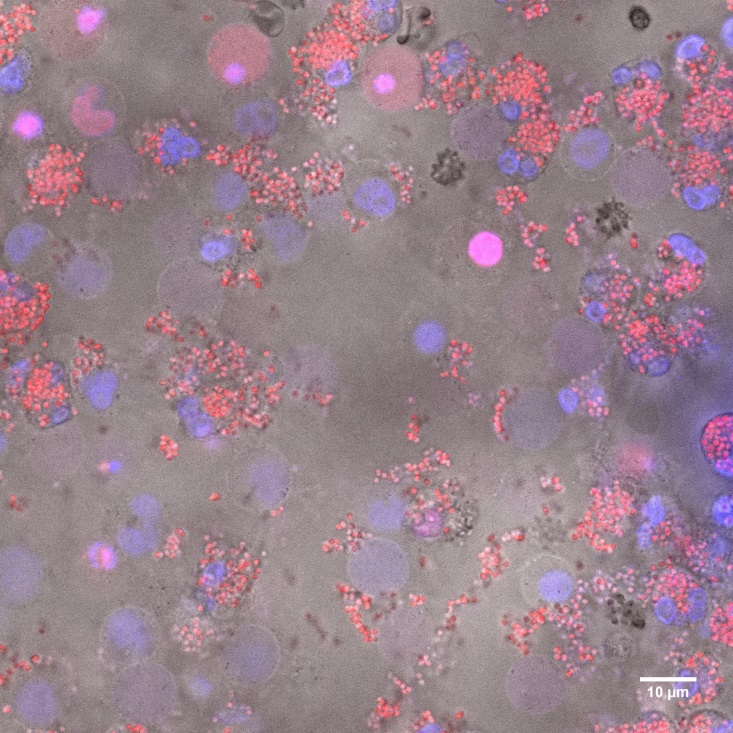
*

**S1 Fig C:** Bright field overlay image of neutrophils (blue) with dead bacteria (red) showing phagocytosis.
